# Supplementary material for: Development of chemokine network inhibitors using combinatorial saturation mutagenesis
Source: Commun Biol. 2025 Apr 3;8:549. doi: 10.1038/s42003-025-07778-6 (PMC11969024; doi:10.1038/s42003-025-07778-6)
Supplement: Supplementary file 4 — Supplementary Data 2 [file 42003_2025_7778_MOESM4_ESM.zip › SupplementaryData3/scripts/xattune_ci.tar!/./script/analysis/JK/R94_CCL8_HD2CM3085_plot.pdf]

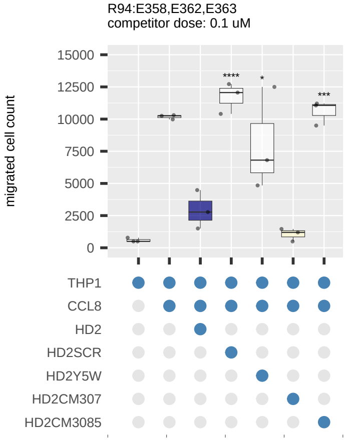

R94:E358,E362,E363
competitor dose: 0.1 uM
migrated cell count
15000
12500
10000
7500
5000
2500
0
****
*
***
THP1
CCL8
HD2
HD2SCR
HD2Y5W
HD2CM307
HD2CM3085
